# G-estimation of causal pathways in vocational rehabilitation for adults with psychotic disorders – a secondary analysis of a randomized trial

**DOI:** 10.1186/s12888-021-03349-1

**Published:** 2021-07-23

**Authors:** Ole Klungsøyr, June Ullevoldsæter Lystad, Helen Bull, Stig Evensen, Torill Ueland, Erik Falkum

**Affiliations:** 1 Department of Research and Innovation, Division of Mental Health and Addiction, Oslo University Hospital, PO Box 4959 Nydalen, 0424 Oslo, Norway; 2 Faculty of Health Sciences, Oslo Metropolitan University, Oslo, Norway; 3 Early Psychosis Treatment, Division of Mental Health and Addiction, Oslo University Hospital, Oslo, Norway; 4 Institute of Clinical Medicine, University of Oslo, Oslo, Norway

**Keywords:** G-estimation, Causal inference, Vocational rehabilitation, Psychotic disorders

## Abstract

**Background:**

Vocational rehabilitation (VR) has increasingly become an important intervention targeting poor occupational functioning in schizophrenia. The Norwegian Job Management Program (JUMP), sought to enhance occupational outcomes by augmenting VR with either cognitive behavioral therapy (CBT) techniques aiming to improve psychotic symptoms or cognitive remediation (CR) aiming to improve cognition. CBT is standard treatment in schizophrenia, but recent meta-analyses question the effect of CBT on negative psychotic symptoms. It is of interest to study the causal role of psychotic symptoms and cognitive functioning on occupational functioning.

**Methods:**

Data from the JUMP VR – program, was reanalyzed with a causal inference method to assess the causal effects of reduced symptoms / improved neurocognitive functioning on occupational functioning measured by number of working hours per week. Participants (*N* = 131) had been randomized to either VR + CBT (*N* = 68) or VR + CR (*N* = 63). Large improvements in number of working hours were demonstrated in both intervention groups (nonsignificant group difference). G-estimation was used to assess the strength and nature of the causal effects, adjusted for time-varying confounding and selection – bias from loss to follow-up.

**Results:**

Significant causal effects of reduction in each of four dimensions of symptoms and improved neurocognition respectively, on number of working hours were found (separate models). The effect of negative symptoms was the strongest and increased in magnitude during the whole observation period, while the effect of two other symptoms and neurocognition was constant. Adjusted for confounding (including potential feedback), the causal effect of a hypothetical change in negative symptoms equal to the average improvement in the CBT group corresponded to an increase in working hours of 3.2 h per week (95% CI: 1.11, 5.35).

**Conclusion:**

High performance of g-estimation in a small psychiatric data set with few repeated measures and time-varying confounding and effects, was demonstrated.

Augmented vocational rehabilitation showed causal effects of intervention targets with the strongest and increasing effect from negative symptoms on number of working hours.

Combination of therapy and activation (indirect and direct approach) might explain improvement in both cognition and negative symptoms, and shed some light on effective ingredients for improved treatment of negative symptoms.

**Supplementary Information:**

The online version contains supplementary material available at 10.1186/s12888-021-03349-1.

## Background

Schizophrenia is associated with positive and negative symptoms, neurocognitive impairment and poor occupational functioning [1, 2]. While positive symptoms (hallucinations, delusions, thought disturbances and impaired reality testing) tend to diminish over time and respond well to medication, this is not the case for negative symptoms (social withdrawal, apathy, avolition and fatigue) [3, 4]. There is a growing consensus that targeting negative symptoms is essential to improve long-term functioning (employment, education, friendships) [5]. However, they are not easy to improve [6]. Recent reviews show less than convincing effects of cognitive behavioral therapy (CBT), one of the most frequently used strategies [5, 7]. In a systematic search of the literature from 2015, the authors conclude that it is necessary to disentangle effective treatment ingredients to make further improvements [4].

Neurocognitive deficits have large impact on functional outcomes like occupational attainment, with no effective pharmacological treatment [8]. In a meta-analysis, cognitive remediation (CR), targeting the patient’s attention, memory and executive functioning, showed stronger effects (small to moderate) on neurocognition when combined with vocational rehabilitation [9]. Cognitive remediation has also been found to improve negative symptoms [10] although this symptom complex is not traditionally a target for CR interventions.

Vocational rehabilitation (VR) in schizophrenia is an approach aimed at helping individuals attain and maintain work. Evidence-based approaches in VR exist [11], but challenges remain causing discontinuation of employment. Thus, in an attempt to strengthen effects, VR programs are being augmented with other therapeutic approaches. Since both symptoms and neurocognitive impairment strongly influence occupational functioning, these factors were targeted in the Job Management Program (JUMP). The JUMP Study was a randomized, multisite hybrid VR program for adults with psychotic disorder in Norway [12–14]. Significant improvements in occupational outcomes were found in both groups (VR + CR versus VR + CBT) across an observation period of two years, with a non-significant group difference [15–17]. Also, both neurocognition and symptoms improved, so far best documented with respect to neurocognitive measures [12, 14]. An association was found between positive change in neurocognition and subsequent occupational functioning, but quantifying a potential “causal” effect of improved neurocognition (or improved symptoms) on occupational functioning has thus far not been explored.

Causal inference - estimating causal effects has become a large field in statistics, with applications in most applied sciences. Causal interpretation of results of a statistical analysis relies on strong assumptions, which are explicitly stated in causal inference methods, in contrast to analysis of associations, with potential misleading interpretation. Causal inference methods are useful, both in observational studies and randomized trials. In an observational study with interest in the effect of a time-varying exposure on an outcome, the proper causal method successfully adjusts for time-varying confounding in cases where traditional methods fail. The same method applies to a randomized trial where the causal effect of interest does not involve the randomized groups, or there is potential selection-bias from differential loss-to-follow-up. G-computation [18], g-estimation for Structural Nested Models [19, 20] and inverse probability weighting (IPW) for Marginal Structural Models (MSMs) [21–23] were proposed by James Robins to overcome difficulties with standard regression methods. The use of IPW estimation for MSMs is widespread, popularized in epidemiology two decades ago, with a considerable amount of citations [23], although applications in psychological / psychiatric research are scarce [24–26]. The less known method of g-estimation for structural nested models [19, 20, 27] is older, but is far less cited than IPW. Even though g-estimation seems complex, it out-performs IPW methods in several ways; higher efficiency, more robust for some forms of bias, more suited for the analysis of continuous exposures and, in contrast to IPW methods it can accommodate effect modification by time-varying covariates [28]. G-computation is even more efficient, but is highly computer intensive with many parametric assumptions (distributional assumptions on confounders etc) which makes it less robust, and can also not accommodate effect modification by time-varying covariates [29]. In an attempt to remedy it’s “underuse”, methodological experts promoted g-estimation for structural nested models in 2014 with a call for more applications [30], and again in a more popularized form in 2016 [28].

This study is a re-analysis of the data from the JUMP program in Norway, with the aim of estimating causal effects of reduced symptoms and improved neurocognition on number of working hours per week using g-estimation. Assessment of how much of the increase in working hours per week can be attributed to improvement of symptoms / neurocognition, can possibly contribute in the design of better VR programs and also to the debate on how to target different core symptoms of schizophrenia.

## Methods

### Sample

The present sample is based on the sample from JUMP, a multisite VR program for adults with psychotic disorders in Norway (*N* = 131). It was a joint venture between health and welfare services with the goal of enhancing occupational outcomes for persons with schizophrenia spectrum disorders. All counties in Norway were invited to participate and six were included in the study. Each county was randomized to one of two interventions VR + CBT or VR + CR. A control group without the augmentation proved impossible to include. More details of design and recruitment of participants are described elsewhere [13]. The intervention consisted of an 10-month extensive VR program with competitive or sheltered work through collaboration between mental health and vocational services, employers and employment specialists in addition to either CR or CBT components. CR and CBT were carried out by employment specialists based in sheltered workshops. Participants had CBT or CR sessions with the employment specialist two hours weekly, and the employment specialists themselves received training (40 h) in CBT or CR, followed by weekly supervision by an experienced health professional [13, 14]. Each employment specialist served around 10 participants, allowing for close collaboration with all involved parties. Participants were assessed at baseline, at the end of the intervention (10 months after baseline) and at approximately 2 years after baseline. The groups are denoted CBT and CR from here onwards. The JUMP study was approved by the Regional Committee of Medical Research Ethics and the Norwegian Data Protection Authority. All participants provided written informed consent.

### Assessments

#### Clinical measures

Clinical assessment was carried out by trained clinicians. Diagnostic evaluation was done with M.I.N.I PLUS [31]. Current level of psychotic symptoms at three time-points, were rated using the Structural Clinical Interview of Positive and Negative Syndrome Scale (SCI-PANSS) [32], measuring three separate dimensions, and one total score: negative symptoms (denoted by *PANSSneg*_*t*_), positive symptoms (*PANSSpos*_*t*_), general symptoms (*PANSSgen*_*t*_), and total score which is a sum of the other three (*PANSSsum*_*t*_). Each dimension was of interest separately (in addition to the total) because of difference in time-course, and specific focus on the challenge with negative symptoms from the literature.

#### Neurocognitive measure

Assessment of neurocognition at three time-points was based on “The Measurement and Treatment Research to Improve Cognition in Schizophrenia” (MATRICS) Consensus Cognitive Battery (MCCB) [33]. Nine out of ten of the MCCB subtests were included in the JUMP protocol (measure of social cognition was excluded), measuring six domains: Speed of processing, Attention/Vigilance, Working memory, Verbal learning, Visual learning and Reasoning and Problem solving [15]. A modified MCCB neurocognitive composite score was calculated as the mean of the nine demographically corrected domain T-scores (denoted by *COGN*_*t*_) [15, 34]. The choice to limit the neurocognitive measure to the mean composite score, was pragmatic. The different domains all had a similar time-course and were found sufficiently represented by the mean, to describe a crude neurocognitive measure.

#### Other covariates

Time-constant variables recorded at baseline consisted of: age, gender, indicators for low education, living alone, marital status, group (*CR*/*CBT*) and history of unemployment, and log-dose of daily medication at baseline. In addition to SCI-PANSS symptoms and neurocognitive MCCB composite score, measure of psychosocial functioning - Global Assessment of Functioning – both function and symptoms were assessed at all three timepoints (*GAFF*_*t*_, *GAFS*_*t*_).

#### Outcome

The outcome of interest in the present application was number of working hours per week, as recorded by the employment specialists at three time-points (denoted by *WH*_*t*_). Both competitive and sheltered work were considered. The latter category is financed partly by e.g. disability benefits, but with similar work demands as in competitive employment. Competitive employment was the goal [13].

### Statistics

### Causal effects in JUMP

Does improvement in psychotic symptoms influence occupational functioning, and if so, how much? This question can be answered by causal inference, which is commonly formalized by means of counterfactuals. In general, with an interest in the effect of an exposure on an outcome, the counterfactual outcome *Y*(*a*) is the potentially unobserved outcome for the hypothetical exposure level *a*. This formalism helps to identify the probability distribution (or functions) of the counterfactuals from observed data. In the following, random variables and their realizations are represented by upper- and lower-case letters respectively.

A conditional average causal effect of a hypothetical intervention (e.g. exposure) setting exposure to *a* versus 0 (0 can represent exposure-free or any reference value) among subjects with covariate value *l*, can be formulated by the linear structural mean model (SMM) [20, 28, 30] as *E*(*Y*(*a*) − *Y*(0)| *l*) = *ψ*^′^*za*, where *E*(.|.) is the conditional population mean, *z* is a covariate vector possibly depending on *l*, and *ψ* is the vector of causal parameters of interest.

For example, with *l* representing gender and *z* = 1, the additive exposure-effect on an outcome from exposure *a* relative to no exposure, is equal between genders. On the other hand, *z* = *l* would describe different exposure effects between genders.

With time-varying exposure, covariates and outcome, the exposure and vector of covariates, *A*_*t*_ and *L*_*t*_, are thought to be assessed at time *t* = 0, 1, 2 with the history up until (and including) *t*, denoted by ${\overline{A}}_t$ and ${\overline{L}}_t$, for example ${\overline{A}}_2=\left\{{A}_0,{A}_1,{A}_2\right\}$. *L*_*t*_ is thought to precede *A*_*t*_ and both precedes the outcomes they affect. ${Y}_s\left({\overline{a}}_t,0\right)$ is the counterfactual outcome at time *s* = 1, 2, 3 for an exposure history equal to ${\overline{a}}_t$ up until time *t* and zero thereafter. This construct facilitates assessments of a causal effect of a time-varying exposure on the following outcome as well as on all subsequent ones, formulated by the structural nested mean model (SNMM) [20]:

1 $$ E\left({Y}_s\left({\overline{a}}_t,0\right)-{Y}_s\left({\overline{a}}_{t-1},0\right)|{\overline{a}}_{t-1},{\overline{l}}_t\right)={\psi}^{\prime }{z}_{st}{a}_t $$

where *s* > *t*. The treatment effect posited in (1) represents the difference in outcome when exposure at time *t* is set equal to *a*_*t*_ and zero thereafter, relative to when exposure at time *t* and onward is set equal to zero, conditional on exposure and covariate history. In other words, the contribution to the exposure effect from a specific time-point (so called “blip”) when later contributions are “removed”.

The causal effects of interest in the present application, is the effect of SCI-PANSS symptoms at time *t*, or the neurocognitive MCCB composite score at time *t*, on number of working hours per week *WH*_*s*_, *s* > *t*. With one dimension of SCI-PANSS symptoms (for example *PANSSneg*_*t*_) considered as “exposure”, then *COGN*_*t*_ is assessed as a potential effect-modifier, and vice versa. The structural nested mean model (SNMMs) with g-estimation is particularly well suited for estimating an effect of a time-varying continuous exposure, and to assess effect-modification by time-varying covariates [28].

Fitting the SNMM for the causal effect of the time-varying *PANSSneg*_*t*_ (or *COGN*_*t*_) on *WH*_*s*_, *s* > *t* is done in three steps (Appendix in supplementary file):

1. Regressing the exposure *PANSSneg*_*t*_ (*COGN*_*t*_) on its history, baseline and preceding time-varying covariates and outcome for each *t* (e.g linear regression), including non-linearities and interactions. The fitted value from this regression is called “propensity score”, denoted *P*_*t*_
2. Regressing the observed outcome *WH*_*t*_ on confounders as well as the terms with previous exposure *z*_*t*, *t* − 1_*a*_*t* − 1_ and propensity score *z*_*t*, *t* − 1_*p*_*t* − 1_ in a repeated measures regression model (GEE with independence working correlation). The *z*_*t*, *t* − 1_*a*_*t* − 1_ coefficient is the preliminary estimate ${\hat{\psi}}^{(0)}$
3. Predicting ${Y}_s\left({\overline{A}}_t,0\right)$ for all *t*, *s* with *s* > *t* by means of ${\hat{\psi}}^{(0)}$. The updated and improved ${\hat{\psi}}^{(1)}$ is found by a second independence GEE with these predictions as outcomes and similar covariates as above. Standard errors are estimated by bootstrap

**Fig. 1:**
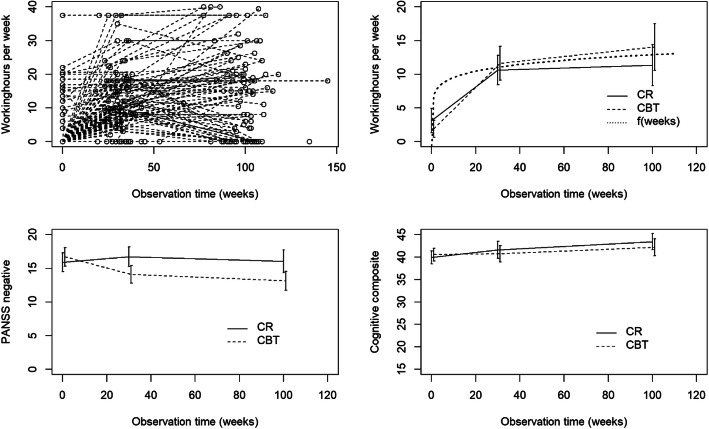
Number of working hours per week (*WH*_*t*_), individually (top left), group means (CBT, CR) for baseline, 30 weeks (mean intervention length), and 100 weeks (mean follow-up time) as well as *f*(*t*) from eq. (3) with *λ =* 0.2 (top right), group means of PANSS negative symptoms (*PANSSneg*_*t*_, bottom left) and neurocognitive composite score (*COGN*_*t*_, bottom right) for baseline, 30 and 100 weeks, 131 adults with broad schizophrenia spectrum disorders in Norway

Different causal hypotheses can easily be assessed with different choices of *z*_*st*_ in (1) (Appendix in supplementary file). To differentiate between short- and long-term effects, and how a long-term effect may decrease over time may be of interest. Different short-term effects allowing for group difference in the time-varying effect or e.g. effect-modification from *COGN*_*t*_ when *PANSSneg*_*t*_ is exposure, are others. However, lack of power is a limitation with increased model complexity. A simple model with effect-modification from a non-linear function of time proved to have a good fit in the present application, and is given by:

2 $$ E\left({Y}_s\left({\overline{a}}_t,0\right)-{Y}_s\left({\overline{a}}_{t-1},0\right)|{\overline{a}}_{t-1},{\overline{l}}_t\right)={\psi}^{\prime }{z}_{st}{a}_t=\left({\psi}_0+{\psi}_1f(t)\right){a}_t $$

for s=1, 2, 3 *s* > *t*, with the following *f*(*t*):

3 $$ f(t)=\left\{\begin{array}{c}\left({t}^{\lambda }-1\right)/\lambda \kern1.25em \lambda \ne 0\\ {}\mathit{\log}(t)\kern3.5em \lambda =0\end{array}\right. $$

where *λ* > 0 (<0) yields a transformation that increases more (less) rapidly than *log*(*t*). *λ* = 1 yields a linear function of time. In the outcome model (step 2 and 3), *f*(*t*) from (3) was included as one component in *l*_*t*_ with *λ* = 0.2 giving a good fit of *WH*_*t*_ as a function of weeks (*f*(*weeks*), *λ* = 0.2 in Fig. 1) in the area of observations (baseline - 0 weeks, 30 weeks, and 100 weeks).

**Fig. 2:**
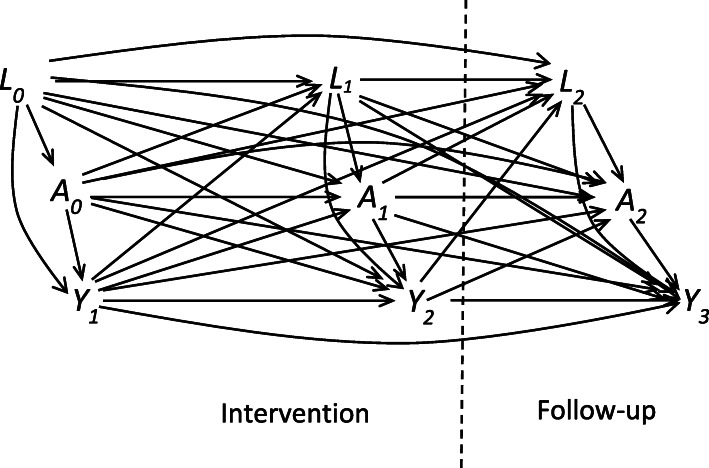
Causal graph (DAG) of design in the JUMP study. *A*_*t*_: PANSS symptoms / neurocognitive composite score, *L*_*t*_: baseline and time-varying covariates, *Y*_*t*_: working hours per week (*WH*_*t*_), 131 adults with broad schizophrenia spectrum disorders in Norway. Indicators *C*_*t*_, for censoring from loss-to-follow up (missing outcome) could be included with arrows entering from preceding exposure, covariates and outcome

A causal graph (DAG) of the JUMP design is shown in Fig. 2.

Three repeated measures of time-varying covariates *A*_*t*_ and *L*_*t*_ and outcome *Y*_*t*_ are shown, and potential direct causal effects between them are indicated with arrows. Both symptoms and cognition are considered fairly stable over time [15] and therefore assumed to precede the outcome (*WH*_*t*_), even though they are assessed at the same time. All the models rely on this unverifiable assumption. With no more than three repeated measures, limited sample size with non-complete data, such a compromise was found necessary. To simplify notation, the index of the outcome is set one higher than the index of the exposure. *L*_*t*_ is made to precede *A*_*t*_ by including only previous confounders (as well as various time-fixed baseline covariates like gender or daily dose of medication). Previous outcome is also included in *L*_*t*_ in the outcome models (step 2 and 3), and allowed to influence both later confounders and exposures. In the DAG (Fig. 2), *Y*_*t*_ is represented separately for illustration.

Measurement error in the exposure variable is not accounted for in the present application. Concerns about reliability in the symptom scores, motivated the attempt to adjust for measurement error in a comparison between the total JUMP sample and a constructed control group (TAU – treatment as usual) [13]. With PANSS measurements as an important confounder for the JUMP / TAU comparison, measurement error would be expected to have an influence. On the other hand, the effect of the symptom scores themselves (as in the present application) on an outcome, would not be influenced in the same way by measurement error (both magnitude and standard error in the coefficient would be expected to increase, leaving the *p*-value approximately unchanged) [36]. Also, it is not obvious how such a measurement error correction should be included in the fitting of the SNMM.

#### G-estimation with multiple imputation in JUMP

Incomplete data were of concern, both for the outcome (*WH*_*t*_) and for different covariates, particularly at the two-year follow-up. Missing values in the outcome was considered to be loss-to-follow-up and the person not allowed to re-enter. Potential selection bias from loss-to-follow-up was adjusted for by inverse probability of censoring weights (Appendix in supplementary file).

To address the issue of incomplete data in covariates, multiple imputation (MI) (Appendix in supplementary file) was performed under the assumption of missing at random (MAR), with the R-package mice [37]. The outcome (*WH*_*t*_) served as covariate (both in exposure and outcome models, steps 1, 2), and was imputed as missing covariate, but not as missing outcome. Out of 34 variables in the data-set, the covariates with missing values were, sorted by decreasing number of missing values (%): *PANSSsum*_3_ – 41 (31.3%), *PANSSgen*_3_ – 40 (30.5%), *PANSSneg*_3_ and *COGN*_3_ – 39 (29.8%), *PANSSpos*_3_ and *WEEKS*_3_ – 38 (29%), *GAFF*_3_ and *GAFS*_3_ – 34 (26%), *PANSSsum*_2_ – 25 (19.1%), *COGN*_2_, *PANSSgen*_2_, *PANSSneg*_2_ and *PANSSpos*_2_ – 23 (17.6%), *WEEKS*_2_ – 21 (16%), *GAFF*_2_ and *GAFS*_2_ – 16 (12.2%), *WH*_3_ – 8 (6.1%), *COGN*_1_ and *PANSSsum*_1_ – 4 (3%), *WH*_2_ and *PANSSneg*_1_ – 3 (2.3%), *PANSSpos*_1_ – 2 (1.5%), *WH*_1_ and *PANSSgen*_1_ – 1 (0.8%). Finally *LowEdu*, *LiveAlone*, *HistUnempl*, *DailyDose*, *Age*, *Gender*, *grp*, *GAFF*_1_, *GAFS*_1_ and *WEEKS*_1_ all had no missing values.

A useful tool for determining potential efficiency gain from MI is the fraction of missing information (*FMI*), approximated by *FMI* = *r*/(1 + *r*) (for a high number of imputations), where *r* is the relative increase in variance due to the missingness [39, 40] (Appendix in supplementary file). The *FMI* is a parameter-specific measure that quantifies the loss of information due to missingness, while accounting for the amount of information retained by other variables [39]. A low number of imputations in MI is usually sufficient, by an argument based on relative efficiency (variance compared to the case with a very large number of imputations). For example, with an *FMI* of 20%, 10 imputations correspond to a relative efficiency above 98% [40].

**Fig. 3:**
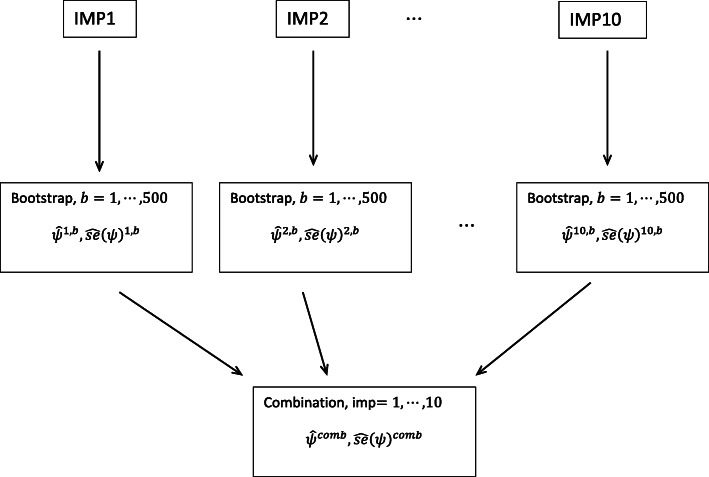
G-estimation set-up in JUMP: Multiple imputation (MI), generating 10 complete datasets, each of which is bootstrapped (500 resamples) to generate parameter-estimate and standard error. The 10 different estimates are combined with Rubin’s rules ([41])

With respect to standard errors in the g-estimation algorithm, the so-called sandwich estimator (in standard regression software) is no longer guaranteed to be conservative, because it ignores the uncertainty in the initial estimate ${\hat{\psi}}^{(0)}$, and therefore the bootstrap is recommended [28]. To account for uncertainty from incomplete data and to achieve unbiased standard errors from the g-estimation, the bootstrap was performed for each imputed dataset. Ten complete datasets were generated from the imputation algorithm, and for each complete dataset a bootstrap parameter estimate with standard error was generated (500 resamples), and finally combined according to Rubin’s rules [41] (Fig. 3 and Table 3).

In accordance with basic bootstrap assumptions, model selection was carried out for every bootstrap sample, both in the censoring weights and propensity score models, to identify the best model. This was solved by the automatic lasso regression algorithm (Appendix in supplementary file) in the R-package glmnet [35]. A large set of covariates was entered for each bootstrap sample, and the lasso algorithm returned the best cross-validated model for that particular sample, in terms of minimum prediction error, while avoiding overfitting.

## Results

The randomization resulted in similar groups (CBT, CR) at baseline. Selected baseline covariates in the total sample and stratified by group are shown in Table 1.

**Table 1:** Selected demographics and baseline covariates

	Total		CBT		CR	
	N	% / mean (SD)	N	% / mean (SD)	N	% / mean (SD)
Gender						
men	92	70.2%	42	61.8%	50	79.4%
women	39	29.8%	26	38.2%	13	20.6%
Age	131	32.72 (7.94)	68	33.24 (8.17)	63	32.15 (7.70)
Low education, high school or less						
No	47	35.9%	27	39.7%	20	31.7%
Yes	84	64.1%	41	60.3%	43	68.3%
Living alone						
No	55	42%	31	45.6%	24	38.1%
Yes	76	58%	37	54.4%	39	61.9%
Marital status– single						
No	25	19.1%	15	22.1%	10	15.9%
Yes	106	80.9%	53	77.9%	53	84.1%
History of unemployment						
No	111	84.7%	59	86.8%	52	82.5%
Yes	20	15.3%	9	13.2%	11	17.5%
Medication, daily dose^a^	131	144.4 (247.9)	68	162.8 (279.1)	63	124.6 (209.6)
SCI – PANSS^a^						
negative	128	16.32 (5.71)	67	16.70 (5.83)	61	15.9 (5.59)
positive	129	13.36 (4.57)	67	12.79 (4.6)	62	13.98 (4.49)
general	130	29.75 (8.28)	67	29.34 (8.89)	63	30.17 (7.64)
sum	127	59.3 (15.43)	67	58.84 (16.55)	60	59.82 (14.19)
MCCB T-score						
Neurocognitive composite	127	40.25 (5.79)	65	40.58 (5.78)	62	39.91 (5.83)

^a^: log-transformed in analysis for less skewness

The total sample (and both groups) consisted of more than 60% men, mostly with lower levels of education, living alone and single. The most pronounced group difference was found for gender (significant), with the highest proportion of women in the CBT group.

To illustrate the time-course of the key variables, mean *PANSSneg*_*t*_ (to represent symptoms), *COGN*_*t*_ and *WH*_*t*_ over time are plotted (Fig. 1). The individual plots of *WH*_*t*_ show large variation, both cross-sectionally and over time (Fig. 1, top left), but with an increasing trend for group-means (Fig. 1, top right) where the mean intervention length of 30 weeks and mean follow-up time of 100 weeks were chosen as time-points. In both groups, there was a large increase in *WH*_*t*_ during the intervention, followed by a smaller increase in the post intervention period. The CBT group had the largest increase, with 3.3 h more on average than the CR group (non-significant group difference). Also plotted is the function *f*(*t*) from (3), used in the outcome model (step 2,3) as one component of *l*_*t*_, representing a good fit of *WH*_*t*_ over time in the area of observations (poor fit below 20 weeks has no impact due to lack of observations). The *PANSSneg*_*t*_ decreased markedly in the CBT group, but not in the CR group (significant group difference in favor of the CBT group - data not shown). The CR group, on the other hand, had a slightly better improvement with respect to *COGN*_*t*_ (non-significant group difference).

Different causal models (SNMMs) were fitted to assess long-term effects, group-differences in short-term effects (interactions with exposure or other covariates) and whether *COGN*_*t*_ could play a role as effect-modifier for *PANSSneg*_*t*_ ’s (or other symptom dimensions) influence on *WH*_*t*_ (or vice versa) (Appendix in supplementary file). No such significant effects were found, but few repeated measures with limited observation time, and model complexity probably resulted in a lack of power for one or more of these tests.

In contrast, the simpler SNMM from eq. (2), also modelling a short-term effect with a constant and a time-varying part, showed significant causal parameters for all dimensions of symptoms and for neurocognition. The neurocognitive composite score and the total symptom score had both significant effects, but of different nature. The effect of *COGN*_*t*_ was constant over time with *ψ*_0_ found to be positive and significant (${\hat{\psi}}_0=0.22,p=0.005$) and with no significant time-varying part (*ψ*_1_). In contrast, *PANSSsum*_*t*_ had no significant constant part (${\hat{\psi}}_0=1.55,p=0.6$) and a negative and significant time-varying part (${\hat{\psi}}_1=-0.31,p=0.018$) Final combination estimates from imputations are shown in Table 2.

**Table 2:** Causal effects of composite cognitive measure (*COGN*_*t*_), PANSS symptoms – sum (log(*PANSSsum*_*t*_)) and negative (log(*PANSSneg*_*t*_)) in separate models, on number of workinghours per week (*WH*_*t*_), by g-estimation in JUMP. Parameters refer to *SNMM* = (*ψ*_0_ + *ψ*_1_*f*(*t*))*a*_*t*_ (2), where *a*_*t*_ is *COGN*_*t*_, log(*PANSSsum*_*t*_), or log(*PANSSneg*_*t*_), and *f*(*t*) is the function in eq. (3) with *λ* = 0.8

	COGN_**t**_			log (PANSSsum_**t**_)			log (PANSSneg_**t**_)		
	Estimate	95% CI	p-value	Estimate	95% CI	p-value	Estimate	95% CI	p-value
*ψ*_0_	0.224	0.067, 0.381	0.005	1.55	−4.25, 7.35	0.6	3.59	−0.383, 7.564	0.077
*ψ*_1_	.	.	.	−0.31	− 0.57, − 0.052	0.018	−0.288	− 0.476, − 0.099	0.0029

The specific form of *f*(*t*) had a large impact on the fit (variation in *λ*), with best fit for *λ* = 0.2 in the outcome model (Fig. 1), and *λ* = 0.8 in the SNMM (larger increase) with symptoms as exposure. Of specific interest in this application was *PANSSneg*_*t*_, which turned out to be dominating the time-varying part of the total symptom score. This dimension changed the most during the observation period and had the strongest causal effect on *WH*_*t*_. The parameter estimate for the constant effect of *PANSSneg*_*t*_, was positive and near significant (${\hat{\psi}}_0=3.59,p=0.077$). The parameter estimate for change over time, *ψ*_1_ was negative and significant (${\hat{\psi}}_1=-0.29,p=0.003$) (Table 2). *PANSSpos*_*t*_ changed less over time and had a constant effect on *WH*_*t*_ with a negative and significant *ψ*_0_ (${\hat{\psi}}_0=-3.46,p=0.011$), and with no significant time-varying part (*ψ*_1_). The same was found for *PANSSgen*_*t*_ with a negative and significant *ψ*_0_ (${\hat{\psi}}_0=-4.2,p=0.026$) and no significant time-varying part.

Chances are that the estimates for the four symptom scores have been biased towards zero by measurement error.

The interpretation of the results is that the mean neurocognitive composite and four dimensions of symptoms, all seemed to have separate significant causal effects on number of working hours. The strongest effect was found for *PANSSneg*_*t*_. A positive *ψ*_0_ seemed to indicate that at baseline, higher level of symptoms yielded higher *WH*_*t*_, but this changed during the intervention with a negative *ψ*_1_. Formulated with counterfactuals, a hypothetical intervention that could lower a person’s *PANSSneg*_*t*_ level at baseline corresponding to 0.23 points on the log-scale (equal to the average change in the CBT group over the whole observation period) would lead to a gain of around 3.23 h per week (95% CI: 1.11, 5.35) by the end of follow-up. The causal effect seemed to be strongest during the intervention period, and slightly attenuated in the post-intervention period yet still increasing, in terms of change in *WH*_*t*_ per unit time.

For *PANSSsum*_*t*_ the effect was similar to *PANSSneg*_*t*_ ’s, but with no constant part, and with an increasing effect across the whole observation period, most during the intervention. A hypothetical intervention that could lower a person’s *PANSSsum*_*t*_ level at baseline corresponding to 0.15 points on the log-scale (equal to the average change in the CBT group over the whole observation period) would lead to a gain of around 2.21 h per week (95% CI: 0.37, 4.06) by the end of follow-up.

For *PANSSgen*_*t*_, *COGN*_*t*_ and *PANSSpos*_*t*_ the effects were found to be constant across the whole observation period, with no time-varying part. A hypothetical intervention that could lower a person’s *PANSSgen*_*t*_ level at baseline corresponding to 0.13 points on the log-scale (equal to the average change in the CBT group) would lead to a gain of around 0.54 h per week (95% CI: 0.06, 1.01) by the end of follow-up. In other words, changes achieved during intervention seemed to be upheld during follow-up.

A hypothetical intervention that could increase the level in *COGN*_*t*_ with 2.4 points (equal to the average change in the CR group) would yield a gain of approximately 0.54 h per week (95% CI: 0.16, 0.92) by the end of follow-up.

A hypothetical intervention that could lower a person’s *PANSSpos*_*t*_ level at baseline corresponding to 0.09 points on the log-scale (equal to the average change in the CBT group) would lead to a gain of around 0.3 h per week (95% CI: 0.07, 0.53) by the end of follow-up.

In the MI routine, both prior and subsequent measurements (if available) were allowed in the imputation model to make use of all available information. Also, interactions discovered in preliminary complete-case analysis were included. Estimated *FMI* s were evaluated to be between 3 and 19%, all considerably smaller than the proportion of missing values in the exposure at follow-up. In *PANSSneg*_*t*_, the proportions of missing values were 2.3, 17.6, and 29.8%, and since a time-varying short-term effect was found, all three time-points contributed in estimation of both *ψ*_0_ and *ψ*_1_. Estimated *FMI* s for *ψ*_0_ and *ψ*_1_ were 9 and 18.4% respectively (Table 3), reflecting that other variables succeeded in retaining information for the missing *PANSSneg*_*t*_ values (*FMIs* less than the proportion of missing values), with more challenge for the time-varying part (*ψ*_1_) than the constant part (*ψ*_0_), due to more complexity (with extra shape-parameter). The exposures with constant effect, all had estimated *FMIs* lower than 15.1%. For example, for *COGN*_*t*_ the proportions of missing values were 3, 17.6, and 29.8% (similar to *PANSSneg*_*t*_), and had an estimated *FMI* of 5.5%. This reflects better prediction of the missing *COGN*_*t*_ with considerable amount of information restored. Also, a constant effect (*ψ*_0_) means a simpler imputation model, estimated from all three time-points.

**Table 3:** Bootstrap estimates for each imputed dataset (combination estimate in Table 2 is mean value across imputations, standard error follows from Rubins’s rules

	COGN_**t**_		log (PANSSsum_**t**_)				log (PANSSneg_**t**_)			
	$\kern0.75em {\hat{\psi}}_0^{imp,b}$	$st. error$	${\hat{\psi}}_0^{imp,b}$	$st. error$	${\hat{\psi}}_1^{imp,b}$	$st. error$	${\hat{\psi}}_0^{imp,b}$	$st. error$	${\hat{\psi}}_1^{imp,b}$	$st. error$
Imputation										
1	0.216	0.08	1.839	2.752	−0.294	0.11	3.686	1.901	−0.3	0.086
2	0.207	0.076	−0.151	2.539	−0.194	0.099	3.084	1.791	−0.249	0.076
3	0.212	0.078	2.128	2.687	−0.331	0.11	3.805	1.965	−0.254	0.089
4	0.234	0.078	1.275	2.668	−0.335	0.126	3.704	1.936	−0.317	0.089
5	0.230	0.08	0.057	2.643	−0.245	0.109	2.632	1.897	−0.248	0.081
6	0.245	0.077	2.436	2.894	−0.344	0.128	4.52	2.025	−0.347	0.095
7	0.252	0.077	0.925	2.636	−0.332	0.116	3.055	1.85	−0.266	0.081
8	0.196	0.078	2.233	2.749	−0.326	0.122	4.334	2.078	−0.346	0.097
9	0.237	0.077	1.348	2.778	−0.304	0.122	3.348	1.881	−0.253	0.082
10	0.212	0.076	3.412	2.816	−0.39	0.129	3.735	1.978	−0.297	0.085
*r*^a^	0.058		0.178		0.242		0.099		0.226	
*FMI*^b^	0.055		0.151		0.195		0.09		0.184	

^a^: relative increase in variance due to missingness, ^b^: fraction of missing information

Selection bias from loss-to-follow-up seemed to be negligible, results with and without censoring weights (Appendix in supplementary file) were nearly identical (data not shown).

## Discussion

The augmented VR program JUMP has previously documented cost-effectiveness, improved occupational outcomes and improved levels of apathy and neurocognition [14, 16, 42]. In this study, causal pathways were assessed, by means of g-estimation, to quantify the magnitude and nature of specific effects that were targets in the CBT and CR groups. Significant causal effects from improvement in different symptoms and a neurocognitive composite on number of working hours per week were found.

The effects were all short-term and characterized as either constant across the observation period or increasing in strength, most during the intervention and slightly less post intervention. PANSS negative symptoms had the strongest effect, followed by PANSS total which both had time-varying effects of magnitude 3.2 h per week (95% CI: 1.11, 5.35) and 2.2 h per week (95% CI: 0.37, 4.06) respectively, for a hypothetical increase in the exposure equal to the mean change in the CBT group. The remaining effects were all found to be weaker and constant over time, with the neurocognitive composite representing an effect of magnitude 0.54 h per week (95% CI: 0.16, 0.92), the same magnitude as PANSS general with 0.54 h per week (95% CI: 0.06, 1.01), and lastly PANSS positive symptoms with a magnitude of 0.3 h per week (95% CI: 0.07, 0.53). In sum, of the five effects under study, PANSS negative symptoms represented the strongest, and PANSS positive symptoms the weakest. The difference in nature between effect of PANSS negative (increasing) and positive (constant) symptoms might seem reasonable in light of their time-trajectories and content. Even though the effects may seem small relative to the total increase in working hours per week (10 h), the adjustment for potential feedback means that their unadjusted contribution might be larger.

Some caution with interpretation is necessary. The short-term causal effects are based on the assumption that the exposure can at least partly be viewed as preceding the outcome, even though they have been assessed at the same time (PANSS negative symptoms / neurocognitive composite score are viewed fairly stable [15]). With incomplete data in many variables (including the key exposure variables), efficiency gain was achieved by multiple imputation. However, unbiasedness relies on assumptions of MAR and no model misspecification in both imputation and analysis models, even though g-estimation provides better protection against misspecification than alternative methods. Missing values in the outcome were few, and selection bias from loss-to-follow-up was found to be negligible.

**Fig. 4:**
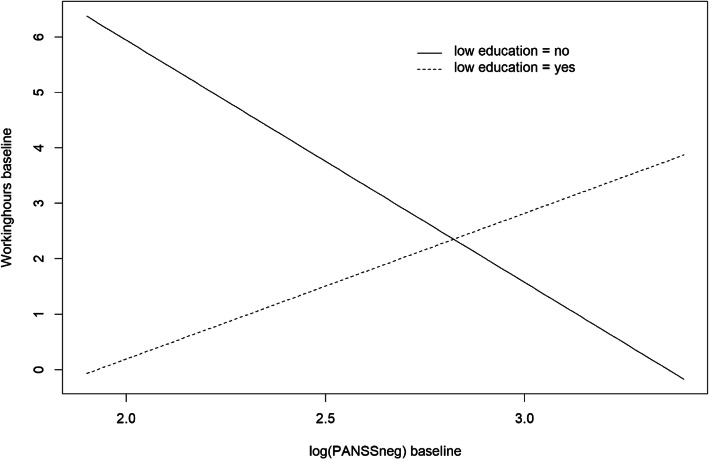
Association between PANSS negative symptoms at baseline (*log*(*PANSSneg*_0_) and initial number of working hours per week (*WH*_1_)

Within the limitations of only three repeated measures, including an intervention period and a limited sample size, g-estimation captured causal information. Few alternative methods are available, particularly with respect to assessment of potential effect-modification by time-varying covariates. Neither PANSS symptoms nor the neurocognitive composite score seemed to play the role of such an effect-modifier, but a non-linear function of time did, which revealed differences in the nature of the causal effects. The results indicated no long-term effect (e.g. a decreasing effect of *A*_*t*_ on *Y*_*s*_ for *s* > *t* + 1). In the causal DAG (Fig. 2) this indicate no arrows from *A*_0_ → *Y*_2_, *A*_0_ → *Y*_3_ or *A*_1_ → *Y*_3_. With more repeated measures, a potential long-term effect would be easier to detect. With respect to the time-varying or constant short-term causal effects, these refer to *A*_0_ → *Y*_1_, *A*_1_ → *Y*_2_ and *A*_2_ → *Y*_3_ in the causal DAG. The PANSS negative effect started out with a near significant positive causal effect at baseline (more symptoms associated with more working hours). Participants working at baseline were either trying to transit from sheltered to competitive employment, or were not coping with current job demands and were in need of support in order to maintain or change current employment [15]. The stratification “low education = yes / no”, showed slopes of opposite signs in the association between PANSS negative symptoms and working hours at baseline (Fig. 4).

This might be explained by reverse causation, the fact that the low education group is over-represented in the sample and that employment perhaps created more negative symptoms for them. In any case, this initial effect changed over time by the intervention (significant interaction with the non-linear function of time). The function of time that gave the best fit indicated that the influence of PANSS negative symptoms was strongest during the intervention, and slightly weaker, but still increasing during the post intervention period. Continuous improvement in PANSS negative symptoms combined with increase in working hours is consistent with a positive feedback mechanism, and interesting with respect to potential long-term impact on occupational functioning.

In terms of magnitude, the effect of PANSS symptoms may have been underestimated due to measurement error. Compromised reliability in the JUMP study in the PANSS measurements was a concern previously, and an attempt to adjust for it was made [13]. In a simple linear regression, the corrected regression coefficient for a covariate with a reliability coefficient of 50%, would be expected to increase two-fold. It’s not clear how to adjust for potential measurement error in the present application.

The causal effects support the choice of targets in the CBT and CR groups. Time-course of PANSS symptoms and neurocognitive composite score stratified on group, shows that each group succeeded best in having an impact on their target measure (Fig. 1, bottom panels). The causal effects of PANSS symptoms or neurocognitive composite are to be interpreted as conditional on each other as well as other covariates. Separate causal effects indicate independent causal pathways through PANSS symptoms and the neurocognitive composite to working hours.

Limitations in the design of JUMP prevents assessment of the causal effect of the CBT or CR intervention. A comparison group, with only the VR part and without CBT or CR, would facilitate this, but was not feasible [43]. How can an improvement in occupational outcome be attributed to for example the CBT intervention, and not simply the “Hawthorne effect”? The causal pathways in the present model can shed some light on this question. The CBT group had a significantly larger improvement on PANSS negative symptoms than the CR group at post intervention. Also, PANSS negative symptoms at post intervention predicted working hours at follow – up. This indicates an indirect effect of the CBT intervention (relative to CR) on working hours (mediation) through PANSS negative symptoms (with some additional assumptions) [44]. Likewise, the CR intervention can have had an indirect effect (relative to CBT) through some other mediator. Both groups had an increase in working hours, but with a difference of 3.3 h in favor of the CBT group (non-significant difference). Three time-points is necessary for mediation analysis to control the time-sequence. Interestingly, with multiple mediators, path-specific effects can be assessed even when the correct sequence of the mediators is unknown [45]. However, using only one time-point for each variable, power to detect different pathways is limited. Lack of power has also probably played a role in the non-significant group × exposure interactions in the present causal models.

This study is the first attempt to assess causal effects in JUMP, where time-varying confounding and potential bias from loss-to-follow-up have been adjusted for and effect modification by time-varying covariates assessed. The large improvement in PANSS negative symptoms in the CBT group is in contrast to the findings in a recent meta-analysis of effects of CBT on negative symptoms in schizophrenia [4]. The authors reviewed the literature and found that beneficial effects of conventional CBT on negative symptoms in schizophrenia from older studies were associated with lower study quality and not supported by more recent studies [46, 47]. The CBT augmentation in JUMP was not conventional CBT, which in fact may provide some explanation, both for the change in negative symptoms and for the improved occupational functioning. The part of negative symptoms that refers to social isolation was effectively reduced by job placement in a social environment. Other negative symptoms accounted for by dysfunctional expectancies [4], could be alleviated by successful matching of the job with the participant’s preference and ongoing support with financial security. In this way, the negative symptoms have been approached both directly and indirectly, distant from the conventional therapy in a therapists’ office. Another recent example of positive effect of CBT on negative symptoms in an indirect way (not included in the above meta-analysis) focused primarily on measures to improve functional outcome (GAF) [48].

Even though a rich body of research on vocational rehabilitation the last decades has contributed to evidence based programs, challenges remain [49]. There is a need for more studies of integrated treatment and vocational rehabilitation [13], to disentangle treatment components to target e.g. negative symptoms [4] and to scale up services in rehabilitation [49].

## Conclusion

High performance of g-estimation in a small psychiatric data set with few repeated measures and time-varying confounding and effects, was demonstrated.

Augmented vocational rehabilitation showed causal effects of intervention targets with the strongest and increasing effect from negative symptoms, on number of working hours.

Combination of therapy and activation (indirect and direct approach) might explain improvement in both cognition and negative symptoms, and shed some light on effective ingredients for improved treatment of negative symptoms.

## Supplementary Information

**Additional file 1. **[20, 28, 40, 41, 35, 50, 51].

## Data Availability

The dataset used in the current study is available from the corresponding author on reasonable request

## Abbreviations

CBT: Cognitive Behavioural Therapy
COGN: Composite measure of neurocognition
CR: Cognitive Remediation
FMI: Fraction of Missing Information
GEE: Generalized Estimating Equations
JUMP: Norwegian job management program for patients with psychotic disorders
MAR: Missing At Random
MSM: Marginal Structural Model
MI: Multiple Imputation
PANSSneg: Structural Clinical Interview of Positive and Negative Syndrome Scale
SMM: Structural Mean Model
SNMM: Structural Nested Mean Model
VR: Vocational Rehabilitation
WH: Number of working hours per week
