# Supplementary material for: G-estimation of causal pathways in vocational rehabilitation for adults with psychotic disorders – a secondary analysis of a randomized trial
Source: BMC Psychiatry. 2021 Jul 23;21:370. doi: 10.1186/s12888-021-03349-1 (PMC8305512; doi:10.1186/s12888-021-03349-1)
Supplement: Supplementary file 1 — Additional file 1. [20, 28, 40, 41, 35, 50, 51]. [file 12888_2021_3349_MOESM1_ESM.docx]

# Appendix

## A1. G-estimation in linear structural nested mean models (SNMMs)

A linear structural mean model (SMM) [20, 28, 30] is a model for the conditional causal effect (conditional on covariates) of any variable of interest (e.g treatment or exposure) on an outcome. It’s linear in an unknown (finite dimensional) parameter $\psi$, which quantifies the causal effect of a hypothetical level of the exposure on a counterfactual outcome, denoted $Y\left( a \right)$ (potentially unobserved outcome for the hypothetical exposure level $a$). A basic assumption in causal inference is that $Y\left( a \right)=Y$ for those that actually had exposure level $A=a$ (consistency). A linear SMM may be formulated as

$E\left( Y\left( a \right)-Y\left( 0 \right) | l \right)=\psi^{'}za$ (A1)

which describes the effect of setting the exposure to $a$ versus 0 (by some intervention) within subjects with covariate values $l$, on the additive scale, where 0 is some reference value (not necessarily “no exposure”), and $z$ is a covariate vector that may depend on (e.g be a subset of) $l$. This is the “point exposure case”, which is the building block for the general case.

The SMM is fitted in two steps: First, the exposure is regressed on a vector of covariates $L$. Let the “propensity score” $P$ be the fitted value from this regression (e.g a linear regression), whether the exposure is continuous or categorical (even though the term is usually reserved for a dichotomous exposure). Secondly, regress the outcome on the vector of covariates $L$, and the terms $ZA$ from the SMM, and $ZP$ where the exposure $A$ has been replaced by the propensity score, given by

$E\left( Y | l,a \right)=\beta_{0}+\beta_{1}^{'}l+\beta_{2}^{'}zp+\psi^{'}za$ (A2)

The estimated causal parameter $\psi$ is equivalent (in large samples) to the g-estimator [28] and has nice properties, like “double-robustness” [28].

The generalization to conditional causal effect of a time-varying exposure, with time-varying covariates and outcome is based on the linear structural nested mean model (SNMM) [20]. The exposure and covariates $A_{t}$ and $L_{t}$ are thought to be assessed at $t=0,\cdots,T-1$ with the history up until time $t$ denoted by $\bar{A}_{t}$ and $\bar{L}_{t}$. The SNMM can be formulated as

$E\left\{ Y_{s}\left( \bar{a}_{t},0 \right)-Y_{s}\left( \bar{a}_{t-1},0 \right) | \bar{a}_{t-1}{,\bar{l}}_{t} \right\}=\psi^{'}z_{st}a_{t}$ (A3)

Where $s>t$, and $Y_{s}\left( \bar{a}_{t},0 \right)$ is the counterfactual outcome where the exposure is set to $\bar{a}_{t}$ up until time $t$, and zero thereafter. This construct fascilitates the estimation of, for each observed outcome $Y_{s}$ at timepoint $s$, not only the causal effect of the exposure immediately preceding the outcome (time $s-1)$, but also earlier exposures (times $s-2\ldots)$ for which the effect of later exposures are subtracted from the outcome. This way the SNMM can be broken down into a sequence of SMM’s and fitted in the same way as in the point exposure case.

First, the exposure on each time $=0,\ldots, T-1$ , is regressed on the history of exposures $\bar{A}_{t-1}$, and covariates $\bar{L}_{t}$, for example in a linear regression, and the fitted value from this regression, called the propensity score, is given by $P_{t}$

$E\left( A_{t} | \bar{a}_{t-1}{,\bar{l}}_{t} \right)=\gamma_{0}+\gamma_{1}a_{t-1}+\gamma_{2}^{'}l_{t}$ (A4)

The causal effect of the exposure at each time on the subsequent outcome is found by viewing the data as a sequence of point-exposure SMMs, with the consistency assumption that $Y_{t}\left( \bar{a}_{t-1} \right)$ equals $Y_{t}$ $t=1,\cdots,T$ for those with observed exposure history $\bar{A}_{t-1}=\bar{a}_{t-1}$, and with the univariate outcome regression in (A2) replaced with a repeated measures regression model like the Generalised Estimating Equations model (GEE) with independence working correlation [28]. The history $\bar{A}_{t-2}$ and $\bar{L}_{t-1}$ are considered baseline covariates (possibly different than the vector of covariates in the exposure regression) and conditioned on, as well as the sequence of propensity scores $P_{t-1}$

$E\left( Y_{t} | \bar{a}_{t-1}{,\bar{l}}_{t-1} \right)=\beta_{0}+\beta_{1}\bar{a}_{t-2}+\beta_{2}^{'}\bar{l}_{t-1}+\beta_{3}^{'}z_{t,t-1}p_{t-1}+\psi^{'}z_{t,t-1}a_{t-1}$ (A5)

This yields the preliminary estimate $\hat{\psi}^{(0)}$. To estimate the effect of $A_{t-2}$, $A_{t-3}\ldots$ again each one is considered a point exposure with the predictions of $Y_{s}\left( \bar{A}_{s-2},0 \right)$ , $Y_{s}\left( \bar{A}_{s-3},0 \right)\ldots$ as outcomes. The unbiased prediction of $Y_{s}\left( \bar{A}_{t},0 \right)$, here denoted by $H_{st}$ for arbitrary $s$ and $t$, $s>t$, is found by the use of $\hat{\psi}^{(0)}$ to subtract the cumulative effect of observed exposure past time $t$ from the observed outcome

$H_{st}=Y_{s}-\sum_{u=t+1}^{s-1} \hat{\psi}^{(0)}'Z_{su}A_{u}$ (A6)

The updated and improved estimate of $\psi$ is found by considering $H_{st}$ for all $s$ and $t$ as repeatedly measured outcomes, with a new independence GEE in the model

$E\left( H_{st} | \bar{a}_{t}{,\bar{l}}_{t} \right)=\beta_{0}+\beta_{1}\bar{a}_{t-1}+\beta_{2}^{'}\bar{l}_{t}+\beta_{3}^{'}z_{st}p_{t}+\psi^{'}z_{st}a_{t}$ (A7)

which yields $\hat{\psi}^{(1)}$. Standard errors by bootstrap are recommended [28].

The structural nested mean model (SNMMs) [20] with g-estimation is particularly well suited for estimating an effect of a time-varying continuous exposure, and to assess effect-modification by time-varying covariates, not possible with the more popular MSM [28].

Different causal hypotheses can easily be assessed with different SNMMs. In the application presented, some candidates were:

$E\left( Y_{s}\left( \bar{a}_{t},0 \right)-Y_{s}\left( \bar{a}_{t-1},0 \right) | \bar{a}_{t-1},\bar{l}_{t} \right)=\psi^{'}z_{st}a_{t}=\left( \psi_{0}I\left( s=t+1 \right)+\psi_{1}\left( s-t-1 \right) \right)a_{t}$ (A8)

$E\left( Y_{s}\left( \bar{a}_{t},0 \right)-Y_{s}\left( \bar{a}_{t-1},0 \right) | \bar{a}_{t-1},\bar{l}_{t} \right)=\psi^{'}z_{st}a_{t}=\left( \psi_{0}+\psi_{1}grp+\psi_{2}l_{t}+\psi_{3}grp\times l_{t} \right)a_{t}$ (A9)

$E\left( Y_{s}\left( \bar{a}_{t},0 \right)-Y_{s}\left( \bar{a}_{t-1},0 \right) | \bar{a}_{t-1},\bar{l}_{t} \right)=\psi^{'}z_{st}a_{t}=\left( \psi_{0}+\psi_{1}f\left( t \right) \right)a_{t}$ (A10)

for $s=1,2,3$ $s>t$, and $I\left( . \right)$ is the indicator function, such that $I\left( arg \right)=1$ if $arg=true$.

The SNMM in (A8) differentiates between short- and long-term effects, e.g. how a long-term effect may decrease over time. The SNMM in (A9) and (A10) parameterizes short-term effects. (A9) allows a group difference in effect, time-varying effect-modification from $l_{t}$, and also a group difference in the effect-modification, or models nested within the most general form. The SNMM in (A10) is a special case of (A9) with effect-modification from a function of time.

Other strengths of the SNMMs and the associated method of g-estimation, compared to the MSM and associated IPW, are more efficient effect-estimates and the so-called “doubly robustness” property, which provides some protection against model-misspecification [28].

## A2. Multiple imputation in JUMP

Compared to a “complete case analysis”, both bias reduction and efficiency gain can be achieved by MI. Availability in standard software lead to an increase in applications of MI in medical research after 2005 [38]. An upper limit for the proportion of missing values in key variables has been postulated, but with little evidence to support it [39]. A more useful tool for determining potential efficiency gain from MI is the fraction of missing information ($FMI$), approximated by $FMI=r/\left( 1+r \right)$ (for a high number of imputations), where $r$ is the relative increase in variance due to the missingness given by [39, 40]

$r=\left( 1+\frac{1}{m} \right)\frac{\sigma_{between}^{2}}{\sigma_{within}^{2}}$ (A11)

with $m$ as number of imputations, the between variance is the sample variance of the coefficients across imputations, and the within variance is the sample mean of the estimated variances across imputations. The $FMI$ is a parameter-specific measure that quantifies the loss of information due to missingness, while accounting for the amount of information retained by other variables [39]. If the estimated $FMI$ (in %), say for an exposure effect parameter, is less than the proportion of missing values in the exposure, it means that other variables contain information about this parameter which is recovered by the imputation, even in the case of a high proportion of missing values. However, for a high proportion of missing, unbiasedness in the MI relies heavily on the untestable assumptions of MAR, and on no misspecification in the imputation and analysis models. A low number of imputations in MI is usually sufficient, by an argument based on relative efficiency (variance compared to the case with a very large number of imputations). For example, with an $FMI$ of 20%, 10 imputations correspond to a relative efficiency above 98 % [40].

## A3. Censoring from loss-to-follow-up

To adjust for potential selection-bias from loss-to-follow-up, inverse probability of censoring weighting is possible, under the “missing at random assumption”, which in the SNMM model means that at each time, the missingness in the outcome is independent of future exposures, covariates and outcomes, conditional on exposures, covariates and outcomes measured up to that time [28]. Adjustment for censoring by loss-to-follow-up is achieved by weighting the outcome regression, with inverse probability weights, similar (but not identical) to the weights used in fitting the marginal structural models. If $C_{t}=1$ is an indicator for a person being lost to follow-up by time $t$, and zero otherwise, the “stabilized weights” that are used and with the least variability, expresses the conditional probability of $C_{s}=0, s>t$ (that the person will continue to stay in the study) conditional on exposure and covariate history, divided by the same probability with updated exposure and covariate history [28]

$w_{Tt}=I\left( C_{T}=0 \right)\prod_{s=t+1}^{T} \frac{Pr\left( C_{s}=0 | C_{s-1}=0,\bar{A}_{t-1},\bar{L}_{t} \right)}{Pr\left( C_{s}=0 | C_{s-1}=0,\bar{A}_{s-1},\bar{L}_{s-1} \right)}$ (A12)

The expression in (A12) reduce to 1 if censoring at each time $s>t$ has no residual dependence on exposure and covariate values past time $t$, conditional on $\bar{A}_{t-1},\bar{L}_{t}$. The $C_{t}'s$ could be included in the causal graph (Figure 2) with arrows entering from preceding exposure, confounders and outcome. Since the SNMM already conditions on exposure and confounder history up to time $t$, no further adjustment is needed for not loss-to-follow-up up to time $t$. Therefore, the weights are usually less variable than the weights for the marginal structural model [28]. In the present application the weights for the outcomes $H_{st}$ (in the GEE) take the form:

$$w_{10}=I\left( C_{1}=0 \right)\frac{Pr\left( C_{1}=0 | C_{0}=0,A_{-1},L_{0} \right)}{Pr\left( C_{1}=0 | C_{0}=0,A_{0},L_{0} \right)}$$

$$w_{20}=I\left( C_{2}=0 \right)\frac{Pr\left( C_{1}=0 | C_{0}=0,A_{-1},L_{0} \right)}{Pr\left( C_{1}=0 | C_{0}=0,A_{0},L_{0} \right)}\times\frac{Pr\left( C_{2}=0 | C_{1}=0,A_{-1},L_{0} \right)}{Pr\left( C_{2}=0 | C_{1}=0,\bar{A}_{1},\bar{L}_{1} \right)}$$

$w_{21}=I\left( C_{2}=0 \right)\frac{Pr\left( C_{2}=0 | C_{1}=0,A_{0},\bar{L}_{1} \right)}{Pr\left( C_{2}=0 | C_{1}=0,\bar{A}_{1},\bar{L}_{1} \right)}$ (A13)

$$w_{30}=I\left( C_{3}=0 \right)\frac{Pr\left( C_{1}=0 | C_{0}=0,A_{-1},L_{0} \right)}{Pr\left( C_{1}=0 | C_{0}=0,A_{0},L_{0} \right)}\times\frac{Pr\left( C_{2}=0 | C_{1}=0,A_{-1},L_{0} \right)}{Pr\left( C_{2}=0 | C_{1}=0,\bar{A}_{1},\bar{L}_{1} \right)}\times\frac{Pr\left( C_{3}=0 | C_{2}=0,A_{-1},L_{0} \right)}{Pr\left( C_{3}=0 | C_{2}=0,\bar{A}_{2},\bar{L}_{2} \right)}$$

$$w_{31}=I\left( C_{3}=0 \right)\frac{Pr\left( C_{2}=0 | C_{1}=0,A_{0},\bar{L}_{1} \right)}{Pr\left( C_{2}=0 | C_{1}=0,\bar{A}_{1},\bar{L}_{1} \right)}\times\frac{Pr\left( C_{3}=0 | C_{2}=0,A_{0},\bar{L}_{1} \right)}{Pr\left( C_{3}=0 | C_{2}=0,\bar{A}_{2},\bar{L}_{2} \right)}$$

$$w_{32}=I\left( C_{3}=0 \right)\frac{Pr\left( C_{3}=0 | C_{2}=0,\bar{A}_{1},\bar{L}_{2} \right)}{Pr\left( C_{3}=0 | C_{2}=0,\bar{A}_{2},\bar{L}_{2} \right)}$$

## A.4 The lasso

The lasso [50] is a shrinkage method with high performance with respect to minimizing prediction error in a regression model. Increasing the number of predictors in a regression model usually means less bias and more variance, leading to overfitting. To avoid overfitting, the lasso shrinks the coefficients by minimizing the $\mathcal{l}_{1}$ penalty function, which results in some of the coefficients are set equal to zero when the predictors are found to have small influence. In the linear regression model

$y_{i}=\beta_{0}+\sum_{j=1}^{p} x_{ij}\beta_{j}+\varepsilon_{i}$ (A14) The lasso solution can be written

$\hat{\beta}^{lasso}=\begin{matrix} argmin \\ \beta\end{matrix}\sum_{i=1}^{n} \left( y_{i}-\beta_{0}-\sum_{j=1}^{p} x_{ij}\beta_{j} \right)^{2}$ (A15)

subject to $\sum_{j=1}^{p} \left| \beta_{j} \right|\leq s$

where $s$ is the amount of shrinkage. The optimal $s$ that minimizes prediction error, can be found by cross-validation, so that the data is divided into let’s say 10 equal-sized parts, 9 of which is used to fit a model with a specific shrinkage and one part to calculate the prediction error. When this is repeated for each part, the mean prediction error is a good estimate[51].
